# Exosomal miR-183-5p Shuttled by M2 Polarized Tumor-Associated Macrophage Promotes the Development of Colon Cancer *via* Targeting THEM4 Mediated PI3K/AKT and NF-κB Pathways

**DOI:** 10.3389/fonc.2021.672684

**Published:** 2021-06-25

**Authors:** Shangxin Zhang, Deguan Li, Min Zhao, Fei Yang, Changye Sang, Changhong Yan, Zhenjun Wang, Yongxiang Li

**Affiliations:** ^1^ Department of Gastrointestinal Surgery & Department of General Surgery, First Affiliated Hospital of Anhui Medical University, Hefei, China; ^2^ Department of General Surgery, Yanqing District Hospital (Peking University Third Hospital Yanqing Hospital), Beijing, China; ^3^ Department of Orthopedics, Beijing Yanqing District Hospital (Peking University Third Hospital Yanqing Hospital), Beijing, China; ^4^ Department of General Surgery, Beijing Chaoyang Hospital, Capital Medical University, Beijing, China

**Keywords:** tumor-associated macrophages, colon cancer, exosome, miR-183-5p, THEM4

## Abstract

**Background:**

Abnormal accumulation of macrophages in the colon cancer (CC) contribute to its progression. miR-183-5p has been confirmed as an oncogene in CC and this article explores the effect and mechanism of exosomal miR-183-5p enriched by M2-polarized tumor-associated macrophages (TAM) on CC cells.

**Methods:**

The human macrophage THP1 was induced to M2 polarization through IL-4 and IL-13 treatment. Exosomes in THP1 were isolated through ultracentrifugation, and the miR-183-5p expression in macrophages and exosomes was verified by quantitative reverse transcription-polymerase chain reaction (qRT-PCR). The miR-183-5p inhibitors and mimics were applied to down-regulate and upregulate miR-183-5p in macrophages, respectively. Meanwhile, CC cell lines LoVo and SW480 were treated with the macrophage conditioned medium and exosomes, respectively. CC cells’ proliferation, invasion, and apoptosis were tested by the cell counting kit-8 (CCK-8) assay, colony formation assay, flow cytometry (FCM), Transwell assay, and xenograft assay, respectively. The profiles of thioesterase superfamily member 4 (THEM4), Akt, and NF-κB were compared by Western blotting (WB).

**Results:**

The miR-183-5p level in M2-TAM and M2-TAM-derived exosomes was significantly increased. Meanwhile, M2-TAM and M2-TAM-derived exosomes significantly facilitated CC cell proliferation and invasion and dampened apoptosis. Overexpression of miR-183-5p in M2-TAM aggravated M2-TAM-mediated promotive effects on CC cells, with down-regulating miR-183-5p reversed M2-TAM-mediated tumor-promotive effects. Mechanically, miR-183-5p targeted THEM4 and inhibited its mRNA and protein expression. Overexpressing THEM4 abated miR-183-5p-mediated carcinogenic effects and inactivates Akt and NF-κB pathways in CC cells. Overall, this article elaborated that exosomal miR-183-5p shuttled by M2-TAM mediated Akt/NF-κB pathway to accelerate CC progression through targeting THEM4.

## Introduction

Colon cancer (CC) is among the frequent clinical malignancies with a high incidence, attacking one million people worldwide annually. Its mortality rate ranks fourth among tumor-associated deaths (1, 2), which has seriously threatened patient’s health and quality of life. The occurrence and development of CC are related to the mediation of various inflammatory cells, including macrophages (3). The inflammatory microenvironment mediated by tumor-associated macrophage (TAM) contributes to the progression of malignant tumors (4, 5). Therefore, the study of the functional state and dynamic changes of macrophages in tumors is significant, which it is expected to become an essential target of tumor therapy.

Macrophages are usually divided into M1-polarization and M2-polarization in reaction to the specific immune response they are involved in. M2-polarized macrophages, also known as alternative activated immunosuppressive macrophages, impede inflammation and promote tumor metastasis, invasion and angiogenesis (6). Exosomes are derived from the endocytic system of cells. Under electron microscopy, exosomes are spherical or cup-shaped, with 30-100 nm in diameter, and rich in effector molecules such as messenger RNAs (mRNAs), microRNAs (miRNAs), and proteins. Exosome-derived miRNAs have attracted more attention from researchers due to their powerful function of regulating gene expression (7). MiRNAs are small non-coding regulatory single-stranded RNA with a length of about 19~25 ribonucleotides. Current reports have revealed that multiple miRNAs are implicated in the occurrence and evolvement of human viral infections and tumors (8). The latest research has shown that macrophage-derived exosomes carrying non-coding RNA and immune factors control immune effectors through immunosuppression or immune activation. For example, miR-223 is overexpressed in macrophages-derived exosomes and can be transferred to co-cultured gastric cancer cells. Functionally, miR-223 enhances doxorubicin resistance in gastric cancer cells by abating F-box and WD repeat domain-containing 7 (FBXW7) (9). MiR-21-5p and miR-155-5p are highly expressed in M2-polarized macrophage-derived exosomes, and they are transferred to CC cells through exosomes to down-regulate Brahma-related gene 1, induce CC cell migration and invasion, and respond to the tumor microenvironment (10).

As a member of miRNAs, miR-183 is reported to aggravate CC progression. For instance, miR-183 had significant higher level in CRC tissues compared with that in the than normal adjacent mucosa (P<0.001), and miR-183 also predicted advanced clinical stage, enhanced lymph node metastasis and distant metastasis, and poorer overall survival of CRC patients (11). On the other hand, miR-183 is reported to promote CC progression *via* promoting migration, proliferation, inhibiting autophagy and apoptosis (12–15). And the combination of fluorouracil (FU) and oxaliplatin (OXA) also repressed the proliferation, promoted apoptosis and arrest cells in G0/G1 phrase of HCT116 cells through inhibiting miR-183 and upregulating SOCS3 (16). Guo J et al. claimed that overexpressed miR-183-5p is transferred to macrophages through exosomes in breast cancer cells, and it heightens the secretion of IL-1b, IL-6 and TNF-a through down-regulating serine-threonine protein phosphatase 2 catalytic subunit alpha (PPP2CA), thereby promoting the cancer progression (17). Collectively, we thought that miR-183-5p might also be shuttled by M2-TAM exosomes and promotes the growth of colon cancer cells.

Thioesterase superfamily member4 (THEM4), also known as C-terminal regulatory protein (CTMP), is an endogenous inhibitor of protein kinase B (Akt), which can bind Akt and reduce its phosphorylation to block the downstream signal transmission (18). THEM4 reversely regulates phosphatidylinositol 3-kinase (PI3K)/Akt and thus has certain anti-inflammatory and anti-tumor effects (19, 20). For example, enolase-phosphatase 1 strengthens glioma cell proliferation and migration by targeting THEM4 to activate the PI3K/Akt/mTOR pathway (21). Vitamin D inactivates Akt/NF-κB by directly up-regulating THEM4, inhibiting COX-2-mediated inflammatory response (22). Bioinformatics analysis (http://starbase.sysu.edu.cn/index.php) indicated that miR-183-5p targeted THEM4. Nevertheless, whether miR-183-5p modulates CC by targeting THEM4/AKT remains to be further studied.

Here, we probe the effects of M2-TAM-derived exosomes (M2-TAM-Exo) on CC. In addition, we found that miR-183-5p was enriched in M2-TAM-Exo. Through gain- and loss-of-function assays of miR-183-5p, it was found that miR-183-5p accelerated the progression of CC. Further studies manifested that miR-183-5p targeted THEM4 and activated Akt and NF-κB pathways to aggravate CC development. This study reveals the regulatory mechanism of M2-TAM-Exo on CC and provides a new reference for clinical CC therapy.

## Materials and Methods

### Ethics Approvement

The animal study was reviewed and approved by the Ethics Review Board of Beijing Chaoyang Hospital, Capital Medical University.

### Cell Lines and Cell Culture

Human CC cell lines (LoVo and SW480) and human mononuclear macrophage THP-1 were bought from the National Collection of Authenticated Cell Cultures (Shanghai, China). All cell lines were maintained in the DMEM-F12 medium supplemented with 10% fetal bovine serum (FBS), 100 U/mL penicillin and 100 mg/mL streptomycin and incubated at 37°C with 5% CO_2_. To induce apoptosis of LoVo and SW480 cells, they were treated with 5 μg/ml cisplatin (Sigma) for 24 hours.

### M2 Polarization of Macrophages

In a 10 cm culture dish, 1×10^7^ THP-1 cells were incubated in a complete medium (10 mL) containing 200 ng/mL PMA for 24 hours and then cultured in a fresh complete medium (10 mL) for 24 hours to produce M0 macrophages. For inducing M1 polarization of macrophages, THP-1 cells were cultured in a 10 ml fresh complete culture medium containing 100 ng/mL LPS (Sigma) and 20 ng/mL IFN-γ (Sigma) for 24 hours. For inducing M2 polarization of macrophages, THP-1 cells were cultured in a 10 mL fresh complete culture medium supplemented with 20 ng/mL IL-4(Peprotech) and 20 ng/mL IL-13 (Peprotech) for 72 hours. After polarization, 10^6^ M1-TAM and M2-TAM were cultured in 10 mL serum-free plates. The conditioned medium (CM) was centrifuged at 1300 rpm (200 g) for 5min, and the supernatant was stored in a refrigerator at −80°C.

### Cell Transfection

miR-183-5p mimics and their negative controls, miR-183-5p inhibitors (miR-183-5p-in) and their negative controls were transfected into M2 macrophages with Liposome 3000 (Thermo Fisher Scientific), respectively. LoVo and SW480 cells in the logarithmic growth phase were seeded in 6-well plates (5×10^5^/well). When the cell growth density was 50%-60%, LoVo and SW480 cells were instantaneously transfected with THME4 overexpression plasmids or negative vector using FuGene^®^ reagents (Promega, Madison, WI, USA) referring to the manufacturer’s protocol. After 48 hours of transfection, the cells were collected for further experiments. MiR-183-5p mimics and their negative controls, miR-183-5p-in and their negative controls, THME4 and THME4-NC were purchased from Genepharma (Shanghai, China).

### The Co-Culture Model of LoVo and SW480 Cells With TAM and M2-TAM

We employed a Transwell chamber composed of six wells (0.4 μm in diameter) to establish an interaction model. LoVo/SW480 cells were inoculated in the lower chamber (2×10^5^ cells), while 6×10^5^ TAM or M2-TAM were seeded in the upper chamber. Then, the co-culture model was maintained in an incubator at 37°C, and the culture medium was changed after incubation for 24 hours. Seventy-two hours later, the lower chamber cells were harvested, and the LoVo/SW480 cells were used for further experiments.

### Quantitative Reverse Transcription-Polymerase Chain Reaction (QRT-PCR)

The TRIzol reagent (Invitrogen, Carlsbad, CA, USA) was employed to extract the total RNA in TAM, M2-TAM, M2-TAM-Exo, LoVo cells, SW480 cells. The concentration was quantified using a NanoDropTM 2000 spectrophotometer (Thermo Fisher Scientific, Rockford, IL, USA). Next, the total mRNA was reversely transcribed into cDNA with PrimeScript™ RT Reagent kit (Invitrogen, Shanghai, China). The SYBR GreenPCR Reagent and ABI7500FAST Real-Time PCR instruments were applied for real-time fluorescent quantitative PCR. The reaction conditions include 95°C pre-denaturation for 30 seconds, 95°C denaturation for 5 seconds, 60°C annealing/extension for 30 seconds, a total of 40 cycles. The 2^-ΔΔCt^ method was employed to evaluate the relative expression of miR-183-5p and THEM4. U6 was the endogenous control of miR-183-5p, while GAPDH was that of THEM4. The experiments were conducted three times. Primer sequences were as follows:

miR-183-5p Forward: 5’- TATGGCACTGGTAGAATTCACT -3’;
miR-183-5p Reverse: 5’- ACGCTTCACGAATTTGCGT -3’;
THEM4 Forward: 5’-tccaggttagatgctgcaca-3’;
THEM4 Reverse: 5’-gagtggagctggcaattctg-3’;
U6 Forward: 5’-TGCGGGTGCTCGCTTCGGCAGC-3’;
U6 Reverse: 5’-CCAGTGCAGGGTCCGAGGT-3’;
GAPDH Forward: 5’- CGGAGTCAACGGATTTGGTCGTAT-3’;
GAPDH Reverse: 5’- AGCCTTCTCCATGGTGGTGAAGAC-3’.

### Isolation and Characterization of Exosomes

Exosomes were collected from the supernatant of LoVo and SW480 medium following the manufacturer’s instructions for the ExoQuick precipitate solution (SystemBiosciences, Mountain View, CA, USA). In short, when the cells reached 80-90% confluence rate in a serum-free medium, they were mixed with Exoquick TC exon precipitate solution at a ratio of 5:1 (medium: Exoquick TC exon precipitate solution), and the supernatant was harvested. The mixture was incubated overnight and centrifuged (1500 rpm, 30 min). Then, particles containing exosomes were resuspended in PBS and filtered through a 0.22 μm filter. Extracted exosomes were preserved at -80°C and observed with transmission electron microscopy (TEM, Tecnai, FEI, Hillsboro, OR, USA). Western blotting (WB) was conducted to monitor the exosome surface markers for CD9, CD63, and HSC70.

### Cell Counting Kit-8 (CCK-8) Assay

LoVo cells were inoculated in 96-well plates (2000 cells/well) and cultured at 37°C with 5% CO_2_, and each group had 4 repetitive wells. Then, 90 μL fresh complete medium was added to each well, and 10 μL CCK-8 solution (Dojindo Molecular Technologies, Kumamoto, Japan) was supplemented. After culturing for 3 hours, the absorbance (A) at 450 nm was determined with a microplate reader (MD FlexStation3, Molecular device, California, USA). The growth curve was drawn with the average value of each group of LoVo/SW480 cells as the ordinate and time as the abscissa. The cell growth in each group was observed at 12, 24, 36, 48, 60 and 72 hours. The proliferation detection method for each group of SW480 cells was the same as above.

### Flow Cytometry (FCM)

LoVo/SW480 cells were obtained to make single-cell suspensions, which were then inoculated in a 25cm^2^ culture bottle. After overnight cell adherence, the primary medium was discarded. The experimental group was supplemented with the culture medium containing 0.3% FBS, and the control group was supplemented with the medium containing an equal volume of PBS. The mediums in the two groups were incubated at 37°C with 5% CO_2_ for 24 hours, and then the supernatant was collected and rinsed with cold PBS three times. The cells were harvested and trypsinized with EDTA-free trypsin. The rest procedures were performed according to the instructions of the AnnexinV-PI Apoptosis Detection Kit (Yeasen Biotech Co., Ltd., Shanghai, China). Briefly, 70% ethanol was used for fixing the collected cells, which were washed with PBS for three times. Then, the cells were double-stained with AnnexinV-FITC and PI (Vazyme) for 15 min at 37°C. Cell apoptosis was determined by FCM within 1 hour. The apoptosis detection method of each group of SW480 cells was the same as above.

### Colony Formation Experiment

LoVo and SW480 cells in each group were inoculated into 6-well plates (1×10^3^ cells/well). After two weeks of culture, the medium was discarded. The cells were cleaned twice with PBS and immobilized with 4% paraformaldehyde (10 min). Afterward, 1 mL crystal violet was added to each well, and the colonies with ≥50 cells were counted under the microscope with low magnification.

### Wounding Healing Assay

LoVo and SW480 cells in the logarithmic growth phase were inoculated in 6-well plates. When the cells reached 80%~90% fusion rate, a scratch was made with a 200 μl sterile pipette tip as far as possible perpendicular to the cells. Then, PBS was adopted to clean the floating cells three times. The width of scratches was observed under a microscope and counted as D 0h. The cells were then cultured in the DMEM medium containing 2.5% FBS (37°C, 5% CO_2_, 24 hours) and immobilized with 4% paraformaldehyde. 48 hours later, the width of scratches was observed under a microscope and counted as D 48h.The cells’ migrative ability was evaluated by the ratio of D 48h/D 0h. The less ratio is, the more migrative the CC cells were.

### Transwell Assay

Cell invasion was assessed by Transwell assay. Transwell chambers (8 μm, Corting, NY, USA) were coated with 200 mg/mL Matrigel (BD, Sanjose, USA) and incubated overnight. LoVo and SW480 cells were then added to the serum-free medium in the upper chamber. DMEM (500 μL) containing 10% FBS was maintained in the lower chamber as a chemotactic agent. After 24 hours of incubation, all the uninvaded cells were wiped off using swabs. Matrigel-coated membranes were fastened with paraformaldehyde and then stained with crystal violet solution. The invaded cell number was calculated by phase-contrast microscopy (Olympus, Tokyo, Japan). The test was done in triplicate, and the measurement was made three times.

### Western Blot

The LoVo and SW480 cells or exosomes were collected, lysed with lysis solution (Roche) on ice for 30 min, and the total protein was separated by centrifugation at 14000 rpm for 30 min. Afterward, 50 μg total protein was loaded on 12% polyacrylamide gel and went through 2-hour electrophoresis at 100 V. The separated protein was then transferred to polyvinylidene fluoride (PVDF) membranes. After being blocked with 5% skimmed milk at room temperature (RT) for 1 hour, the membranes were washed with TBST 3 times (10 min each time) and incubated with the antibodies (all from Abcam, MA, USA) of CD9 (ab263019, 1: 1000), CD63 (ab271286, 1: 1000), HSC70 (ab76005, 1: 1000), THEM4 (ab106435, 1:1000), p-AKT (ab38449, 1:1000), AKT (ab8805, 1:500), p-NF-κB (phospho S536) (Ab106435, 1: 1000), NF-kB (ab32536, 1: 1000), and GAPDH (ab8245, 1: 1000) at 4°C overnight. After the membranes were rinsed with TBST, they were incubated with horseradish peroxidase (HRP)-labeled anti-rabbit secondary antibody (concentration 1:300) for 1 hour at RT. Next, TBST was employed to wash the membranes 3 times (10 min each). At last, a Western blotting reagent (Invitrogen) was used for blots imaging, and the gray intensity of each protein was determined *via* Image J.

### Xenograft Tumor Model

One-month-old male BALB/c nude mice were bought from Shanghai Slac Laboratory Animal Co., Ltd. Mice were randomly divided into 3 groups (10 mice in each group) and raised in animal care facilities under specific pathogen-free conditions. Then, M2-TAM, M2-TAM transfected with miR-185-5p mimics (M2-TAM^miR-183-5p^), M2-TAM transfected with miR-183-5p-in (M2-TAM^miR-183-5p-in^) exosomes, and SW480 cells (2 ×10^6^) were co-cultured and subcutaneously injected into the nude mice. The tumor length and width were monitored every other week, and the volume = 0.5× length ×width ^2^. Four weeks later, 5 mice from each group were randomly selected and killed, and their tumors were removed and weighed. The remaining mice in each group were sacrificed with pentobarbital sodium (10 mg/kg body weight), and their lung tissues were collected for histopathological evaluation. All animal tests were authorized by the Animal Care and Use Committee of Beijing Chaoyang Hospital of Capital Medical University.

### Hematoxylin&Eosin (H&E) Staining

The right lung tissues of the above mice were fixed with 4% paraformaldehyde at 4°C for 24 hours, dried with ethanol, and got paraffin-embedded. Next, the tissues were sliced (5 μm thick) with a slicer (Leica RM2125RT, Leica, Nussloch, Germany), and then stained with H&E using the H&E staining kit (Beyotime, Shanghai, China) according to the producer’s instructions. Sections were inspected by an Olympus BX 51 optical microscope (Olympus Corporation, Tokyo, Japan) at 200 × magnification.

### Immunohistochemistry (IHC)

Tumor tissues were immobilized with 4% formalin solution (Beyotime, Shanghai, China) and then paraffin-embedded. The 4 μm-thick sections were prepared. After blocking the endogenous peroxides and proteins and blocked with 5% goat serum, the sections were incubated overnight with the primary antibodies [including anti-Ki67 (ab16667, Abcam, MA, USA) and anti-THEM4 (ab106435, Abcam, MA, USA) monoclonal antibodies)] at 4°C. Then, the sections were cleaned with phosphate buffer saline (PBS), incubated with the horseradish peroxidase (HRP)-conjugated secondary antibody at 37°C for 1 hour, and then stained with 3, 3-diaminobenzidine (DAB) solution for 3 min at room temperature. The nucleus was counted with hematoxylin. Finally, the staining was observed under Olympus BX 51 optical microscope (Olympus Corporation, Tokyo, Japan) at 200 × magnification.

### Statistical Analysis

SPSS software (version 20.0, Chicago, IL, USA) analyzed the statistics. All data were presented as mean ± SD. Two-group data analysis was made by student’s *t* test, and multiple-group data were analyzed by one-way analysis of variance (ANOVA) followed by the Student-Newman-Keuls post-hoc analysis. *P* < 0.05 indicated statistical significance.

## Results

### M2-Polarized Macrophages Enhanced CC Cells’ Proliferation, Migration, and Invasion and Weakened Apoptosis

**Figure 1:**
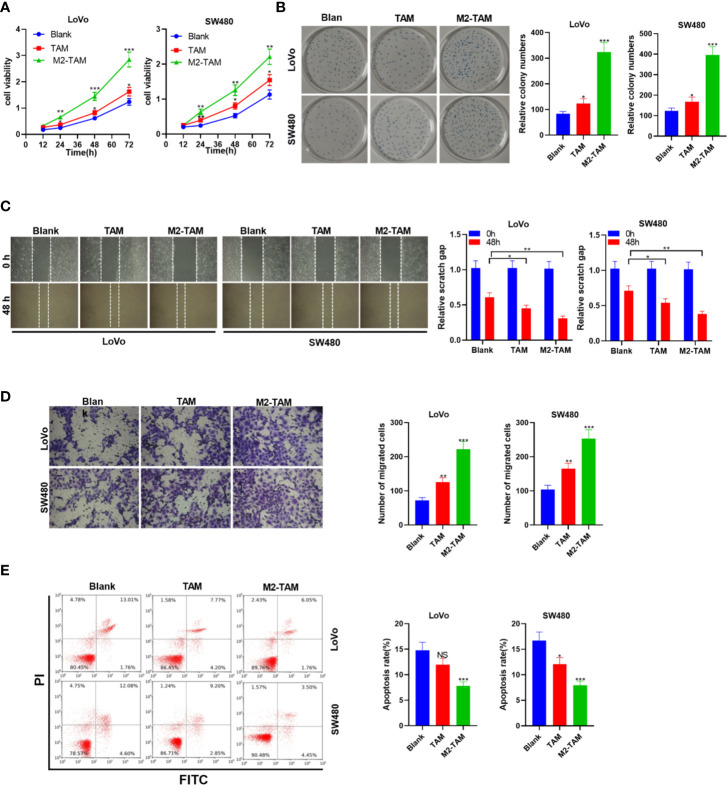
M2-polarized macrophages heightened the proliferation, migration, invasion, and attenuated apoptosis in CC. M2-TAM was co-cultured with LoVo and W480 cells, respectively. **(A)** CCK-8 assay was utilized to verify CC cell proliferation. **(B)** The colony formation experiment was implemented to determine cell colony formation. **(C)** Detection of cell migration by wound healing test. **(D)** Cell invasion was tested by Transwell assay. **(E)** Apoptosis was examined by FCM. ns *P* > 0.05, *P < 0.05, **P < 0.01, *** *P <* 0.001 (*vs*. Blank group). N=3.

Human tumor associated macrophages (TAM) were treated with IL-4 and IL-13 to construct M2-TAM. CC cell lines LoVo and SW480 cells were co-cultured with TAM and M2-TAM to probe the effect of M2-polarized macrophages on CC progression. The experimental groups contained the blank group, TAM group and M2-TAM group. CC cells’ proliferation was examined by the CCK-8 assay and colony formation experiment. The results revealed cell proliferation of the Blank group, TAM group and M2-TAM group showed increased proliferation (*P*<0.05, **Figures 1A, B**). Cell migration and invasion were tested by the wound healing test and Transwell assay, respectively. It could be seen that TAM slightly heightened CC cells’ migration and invasion, while M2-TAM had a stronger effect than that of TAM (*P*<0.05, **Figures 1C, D**). FCM revealed that the apoptosis rate of the Blank group, TAM group and M2-TAM group decreased apoptosis level (*P*<0.05, **Figure 1E**). These findings manifested that M2-polarized macrophages facilitated CC cells’ proliferation, migration and invasion, and weakened apoptosis.

### MiR-183-5p Was Enriched in M2-TAM-Exo

**Figure 2:**
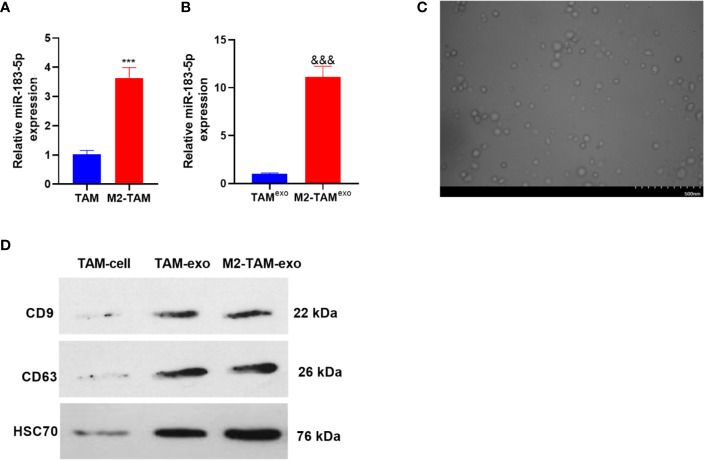
MiR-183-5p was enriched in M2-TAM-Exo. The miR-183-5p expression in M2-TAM was detected. **(A, B)** The profiles of miR-183-5p in TAM, M2-TAM and TAM-Exp/M2-TAM-Exo were compared by qRT-PCR. **(C)** TAM and M2-TAM-Exo were identified by Electron microscopy. **(D)** WB was implemented to verify the exosome surface markers CD9, CD63 and HSC70. ***P < 0.001(*vs*. The TAM group), ^&&&^ *P < *0.001(*vs*. the TAM ^exo^ group). N=3.

The miR-183-5p expression in TAM and M2-TAM was verified by qRT-PCR, and the results manifested that the miR-183-5p level was elevated in the M2-TAM (compared with TAM group *P*<0.05, **Figure 2A**). Further, the miR-183-5p profiles in the exosomes of TAM and M2-TAM were examined, and it was discovered that miR-183-5p expression was higher in M2-TAM-Exo compared with that in TAM (*P*<0.05, **Figure 2B**). M2-TAM-Exo were isolated through differential centrifugation, and the morphology of purified exosomes was observed using TEM. As a result, the exosomes’ diameter was mainly between 30-100 nm (**Figure 2C**). WB results confirmed that both TAM-exo and M2-TAM-Exo has enriched expression of exosome markers CD9, CD63, and HSC70 (**Figure 2D**). The above results indicated that miR-183-5p was enriched in M2-TAM-Exo.

### Upregulation of miR-183-5p in M2-TAM-Derived Exosomes Promoted the Malignant Phenotypes of CC Cells

**Figure 3:**
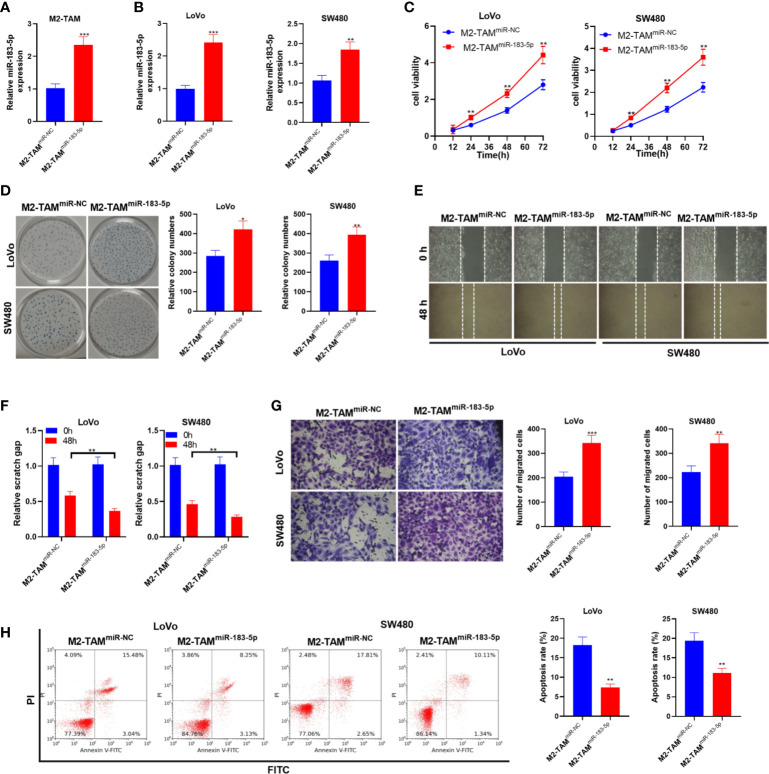
M2-TAM-Exo overexpressing miR-183-5p promoted the malignant phenotype in CC cells. Cisplatin (5 μm) was used for inducing apoptosis of LoVo and SW480 cells. M2-TAM overexpressing miR-183-5p were co-cultured with LoVo and SW480 cells, respectively. **(A)** The miR-183-5p level in M2-TAM was monitored by qRT-PCR. **(B)** The miR-183-5p profile in LoVo and SW480 cells was monitored by qRT-PCR. **(C, D)** CCK-8 assay and colony formation assay were implemented to test CC cell proliferation. **(E)** The ability of cell colony formation was tested. **(F)** Wound healing test detected cell migration. **(G)** Cell invasion was measured by Transwell assay. **(H)** Cell apoptosis was verified by FCM. *P <0.05, **P < 0.01, ***P < 0.001 (*vs*. The M2-TAM ^miR-NC^ group). N=3.

Furthermore, we transfected miR-183-5p mimics into M2-TAM and treated LoVo and SW480 cells with M2-TAM-Exo. Firstly, the miR-183-5p overexpression model was constructed in M2-TAM (*P*<0.05, **Figure 3A**). The miR-183-5p level in LoVo and SW480 cells was monitored by qRT-PCR. It turned out that miR-183-5p was overexpressed in CC cells after co-culturing with M2-TAM-Exo overexpressing miR-183-5p (*P*<0.05, **Figure 3B**). CCK-8 assay and colony formation experiment demonstrated that miR-183-5p overexpression in M2-TAM heightened LoVo and SW480 cell proliferation (*P*<0.05, **Figures 3C, D**). Furthermore, cell migration was examined by wound healing test, and cell invasion was monitored by Transwell assay. It turned out that overexpressing miR-183-5p strengthened LoVo and SW480 cell migration and invasion (*P*<0.05, **Figures 3E–G**). Moreover, FCM testified that the apoptosis rate in the M2-TAM^miR-183-5p^ group was decreased (*vs*. the M2-TAM^miR-NC^ group) (*P*<0.05, **Figure 3H**). These data confirmed that after overexpressing miR-183-5p in M2-TAM, M2-TAM-Exo promoted the malignant phenotypes of CC cells.

### Knocking Down miR-183-5p in M2-TAM Exosomes Suppressed the Malignant Phenotypes of CC Cells

**Figure 4:**
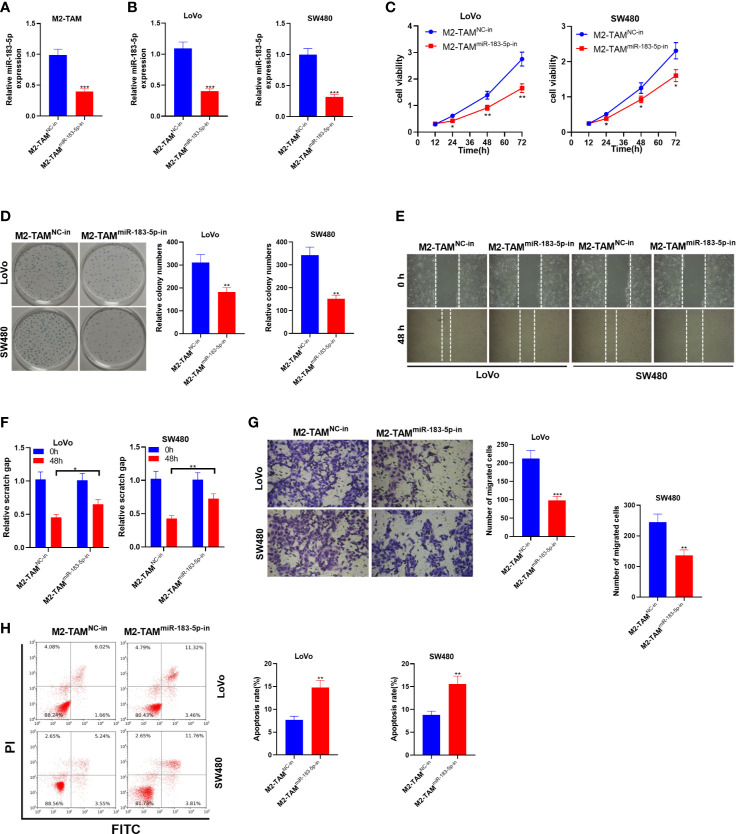
M2-TAM-Exo knocking down miR-183-5p attenuated the malignant phenotype of CC cells. MiR-183-5p-in was transfected into M2-TAM and then co-cultured with LoVo and SW480 cells, respectively. **(A)** The miR-183-5p profile in M2-TAM was monitored by qRT-PCR. **(B)** qRT-PCR verified the miR-183-5p profile in LoVo and SW480 cells. **(C, D)** CCK-8 assay and colony formation assay were implemented to detect CC cell proliferation. **(E)** The ability of cell colony formation was tested. **(F)** Detection of cell migration by wound healing test. **(G)** Cell invasion was measured by Transwell assay. **(H)** Cell apoptosis was examined by FCM. *P < 0.05, **P < 0.01, ***P < 0.001 (*vs*. The M2-TAM ^NC-in^ group). N=3.

Similarly, the miR-183-5p knockdown model was established in M2-TAM, and M2-TAM-Exo were cultured with LoVo and SW480 cells to study the effect of M2-TAM knocking down miR-183-5p on CC cells. Firstly, the miR-183-5p knockdown model was established (*P*<0.05, **Figure 4A**). The miR-183-5p profile in LoVo and SW480 cells was monitored by qRT-PCR. It turned out that miR-183-5p was knocked down in CC cells after the miR-183-5p expression in M2-TAM was inhibited (*P*<0.05, **Figure 4B**). The CCK-8 method and colony formation assay were conducted to verify cell proliferation. As a result, the proliferative ability of LoVo and SW480 cells was decreased after miR-183-5p inhibition (*P*<0.05, **Figures 4C, D**). Transwell assay illustrated that LoVo and SW480 cells’ migration and invasion were abated after miR-183-5p knockdown (*P*<0.05, **Figures 4E–G**). FCM verified that the apoptosis rate of the M2-TAM^miR-183-5p-in^ group was strengthened (*P*<0.05, **Figure 4H**). These results confirmed that M2-TAM-Exo inhibited the malignant phenotype of CC cells after inhibiting miR-183-5p in M2-TAM.

### MiR-183-5p Targeted THEM4

**Figure 5:**
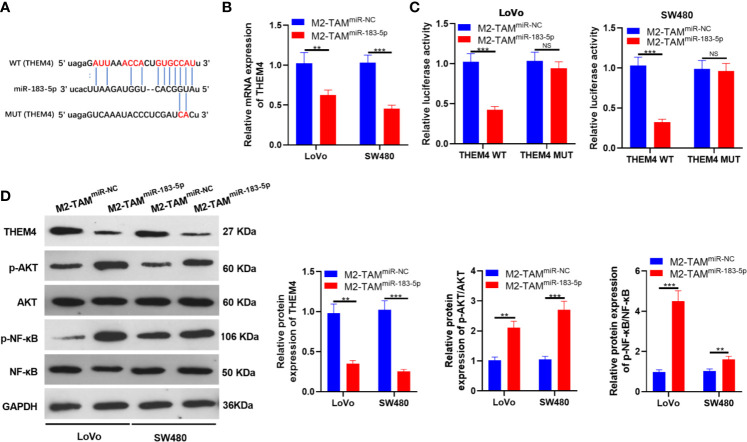
miR-183-5p targeted THEM4. **(A)** Bioinformatics predicted the potential binding sites between miR-183-5p and THEM4. **(B)** The THEM4 level in LoVo and SW480 cells was monitored by qRT-PCR. **(C)** The targeting association between miR-183-5p and THEM4 was determined by the dual-luciferase reporter assay in SW480 and LoVo. **(D)** The expression of THEM4, Akt and NF-κB was compared by WB. ns, *P* >0.05, **P < 0.01, ***P < 0.001 (*vs*. The M2-TAM ^miR-NC^ group). N=3.

We searched the molecular target of miR-183-5p through TargetScan to further explore its downstream regulatory mechanism. As a result, THEM4 was an underlying target of miR-183-5p (**Figure 5A**). M2-TAM-Exo overexpressing miR-183-5p were co-cultured with LoVo and SW480, respectively, and the THEM4 mRNA expression was detected. The results demonstrated that THEM4 was down-regulated after miR-183-5p overexpression (*P*<0.05, **Figure 5B**). Next, we implemented a dual-luciferase reporter assay to confirm whether miR-183-5p bound to THEM4. It turned out that miR-183-5p abated the luciferase activity of THEM4-WT, while it had no impact on THEM4-MUT (*P*<0.05, **Figure 5C**). The THEM4 profile was monitored by WB, and the results manifested that overexpressing miR-183-5p significantly down-regulated THEM4 (*P*<0.05, **Figure 5D**). Meanwhile, WB confirmed that p-AKT and p-NF-κB were up-regulated in the M2-TAM^miR-183-5p^ group (*P*<0.05, **Figure 5D**). The above experiments confirmed that miR-183-5p targeted THEM4 and inhibited its expression.

### Overexpressing THEM4 Abated the Malignant Phenotypes of CC

**Figure 6:**
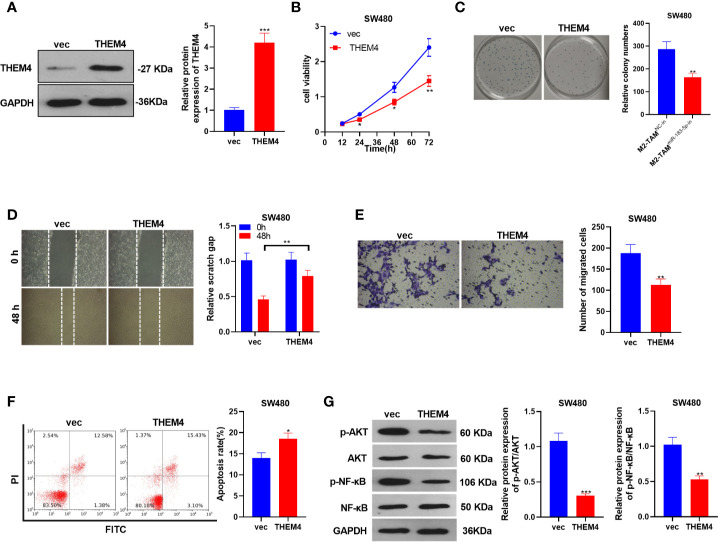
Overexpressing THEM4 dampened the malignant phenotypes of CC. THEM4 overexpression model was established on SW480 cells. **(A)** The THEM4 expression was detected by WB. **(B)** CCK-8 assay was employed to examine CC cell proliferation. **(C)** Colony formation assay was applied to detect the ability of cells to form colonies. **(D)** Wound healing test determined cell migration. **(E)** Cell invasion was monitored by Transwell assay. **(F)** Apoptosis was determined by FCM. **(G)** Akt and NF-κB levels were compared by WB. *P < 0.05, **P < 0.01, ***P < 0.001 (*vs*. The M2-TAM ^miR-NC^ group). N=3.

The THEM4 overexpression model was constructed in SW480 cells, and the influence of THEM4 overexpression on CC cells’ proliferation, migration, invasion and apoptosis was probed (*P*<0.05, **Figure 6A**). SW480 cell proliferation was determined by the CCK-8 method and colony formation assay, and it was found that overexpressing THEM4 inhibited cell proliferation (*P*<0.05, **Figures 6B, C**). The wound healing test and Transwell assay results showed that overexpressing THEM4 hampered SW480 cell migration and invasion (*P*<0.05, **Figures 6D, E**). FCM results manifested that overexpressing THEM4 heightened the apoptosis rate (*P*<0.05, **Figure 6F**). WB was implemented to determine the relative expression of p-AKT and p-NF-κB in each group, and it was found that their expression was down-regulated after THEM4 overexpression (*P*<0.05, **Figure 6G**). These results confirmed that overexpressing THEM4 inhibited the p-AKT and p-NF-κB expression and attenuated the malignant phenotype of CC.

### Overexpressing THEM4 Abated the M2-TAM^miR-183-5p^ Exosomes Induced Malignant Progression of CC Cells

**Figure 7:**
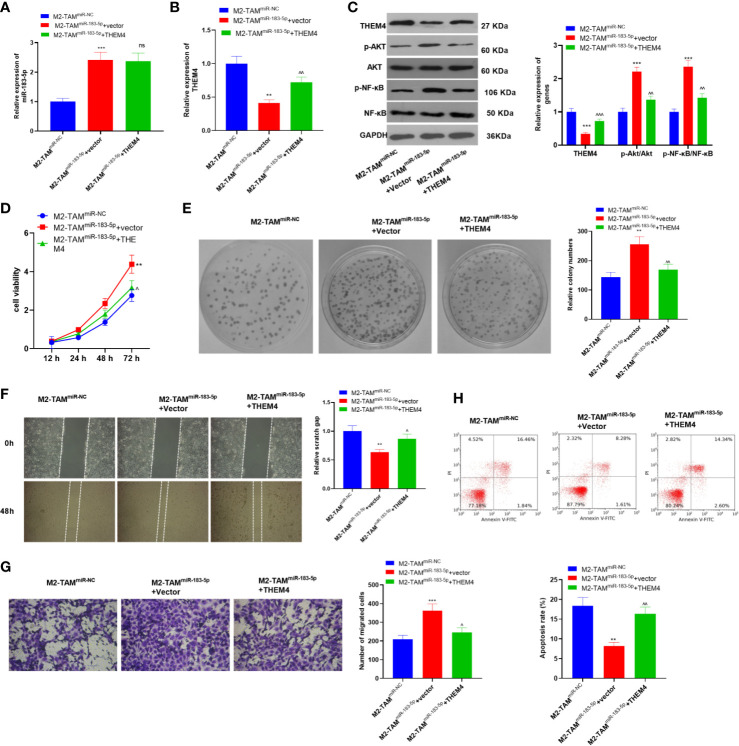
Overexpressing THEM4 abated the M2-TAM^miR-183-5p^ exosomes induced malignant progression of CC cells. The THEM4 overexpression model was constructed in SW480 cells, which were then deal with exosomes from M2-TAM^miR-NC^ or M2-TAM^miR-183-5p^. **(A, B)** The miR-183-5p and THEM mRNA profiles in SW480 cells were monitored by qRT-PCR. **(C)**The THEM4, AKT, NF-κB levels in SW480 cells were detected by WB. **(D)** CCK-8 assay was employed to examine CC cell proliferation. **(E)** Colony formation assay was applied to detect the ability of cells to form colonies. **(F)** Wound healing test determined cell migration. **(G)** Cell invasion was monitored by Transwell assay. **(H)** Apoptosis was determined by FCM. **P < 0.01, ***P < 0.001 (*vs*. The M2-TAM ^miR-NC^ group), ns, *P* > 0.05, ^*P <* 0.05, ^^*P* < 0.01, ^^^*P < *0.001 (*vs*. The M2-TAM ^miR-183-5p^+Vector group). N=3.

The THEM4 overexpression model was constructed in SW480 cells, which were then deal with exosomes from M2-TAM^miR-NC^ or M2-TAM^miR-183-5p^, and the proliferation, migration, invasion and apoptosis were detected. we found that M2-TAM^miR-183-5p^ enhanced miR-183-5p expression in SW480 cells, while repressed THEM4 and inactivated AKT/NF-κB pathway (**Figures 7A–C**). Next, the proliferation was determined by the CCK-8 method and colony formation assay, and it was found that overexpressing THEM4 inhibited cell proliferation promoted by M2-TAM^miR-183-5p^ exosomes (*P*<0.05, **Figures 7D, E**). The wound healing test and Transwell assay results showed that overexpressing THEM4 hampered M2-TAM^miR-183-5p^ exosomes induced SW480 cell migration and invasion (*P*<0.05, **Figures 7F, G**). FCM results manifested that compared with M2-TAM^miR-183-5p^ +Vector group, overexpressing THEM4 heightened the apoptosis rate (*P*<0.05, **Figure 7H**). Collectively, these results confirmed that overexpressing THEM4 inhibited M2-TAM^miR-183-5p^ exosomes induced malignant progression of CC cells.

### miR-183-5p-Enriched M2-TAM Exosomes Strengthened CC Cell Proliferation and Metastasis *In Vivo*

**Figure 8:**
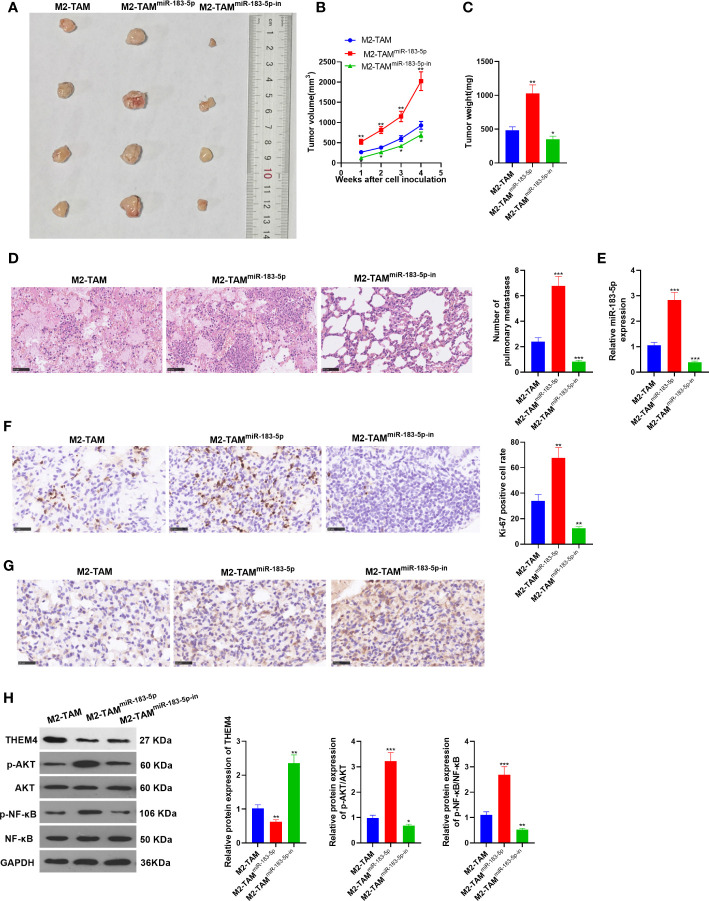
miR-183-5p enriched M2-TAM-Exo heightened the proliferation and metastasis of CC cells *in vivo*. The xenograft model was constructed to observe the effect of miR-183-5p *in vivo*. **(A)** The figure of xenograft tumor. **(B, C)** Tumor volume and weight. **(D)** H&E staining. **(E)** The miR-183-5p profile in the xenograft tumor was determined verified by qRT-PCR. **(F, G)** The levels of Ki67 and THEM4 were compared by IHC. **(H)** The relative expression of THEM4, AKT and p-NF-κB in tumor tissues of each group was tested by WB. *P <0.05, **P < 0.01, ***P <0.001 (*vs*. M2-TAM group). N=3.

The xenograft model was constructed to observe the effect of miR-183-5p *in vivo*. M2-TAM, M2-TAM^miR-183-5p^, and M2-TAM^miR-183-5p-in^ were co-cultured into SW480 cells, respectively, and the co-cultured cells were subcutaneously injected into one-month-old nude mice with a syringe. Four weeks later, the mice were sacrificed. Next, the xenograft tumors were taken, weighed and photographed (**Figure 8A**). As a result, the tumor volume and weight in the M2-TAM^miR-183-5p^ group were higher than those in the M2-TAM group, while the results in the M2-TAM^miR-183-5p-in^ group were the opposite (*vs*. the M2-TAM group) (*P*<0.05, **Figures 8B, C**). H&E staining results showed that miR-183-5p overexpression in TAM elevated the number of tumor metastases in lung tissue, while the miR-183-5p inhibition showed the reverse effects (*P <*0.05, *vs*. the TAM group, **Figure 8D**). In addition, qRT-PCR demonstrated that miR-183-5p had the highest expression in the M2-TAM^miR-183-5p^ group while had the lowest expression in the M2-TAM^miR-183-5p-in^ group (*P*<0.05, **Figure 8E**). Meanwhile, IHC showed that Ki67 and THEM4 were up-regulated in the mice transfected with miR-183-5p and down-regulated in those of transfected with miR-183-5p-in (*vs*. the M2-TAM group, **Figures 8F, G**). WB was conducted to analyze the expression of THEM4, p-AKT and p-NF-κB in each group. As a result, THEM4 was down-regulated, while p-AKT and p-NF-κB were up-regulated after miR-183-5p overexpression. On the contrary, the above molecules’ expression was reversed in the TAM^miR-183-5p-in^ group (*P*<0.05, **Figure 8H**). These results confirmed that miR-183-5p enriched M2-TAM-Exo heightened CC cell proliferation and metastasis *in vivo*.

## Discussion

CC is a common malignancy of the digestive tract, and its therapeutic effect and prognosis are related to many factors. There are more than one million new cases of CC in the world every year. Current studies have found that TAMs are the most abundant cells involved in tumor occurrence and progression, accounting for 50% of the tumor volume (23). An increased amount of TAM (including M1-TAM and M2-TAM) has been found in the intestinal mucosa of many CC patients and animal models. Functionally, TAM promotes angiogenesis, mediates the immunosuppression, and aggravates the inflammatory response in CC (24–26). This study found that M2-TAM heightened CC cell proliferation, migration, and invasion and reduced apoptosis. MiR-183-5p was highly expressed in M2-TAM-Exo. MiR-183-5p activated the PI3K/AKT and NF-κB pathways by targeting THEM4, thereby facilitating CC cells’ proliferation, invasion, and metastasis. This study suggests that inhibiting miR-183-5p is a new therapeutic method for CC treatment.

Exosomes form multivesicular bodies from late endosomes, which then sprout into small vesicles and can be secreted outside the cell (27, 28). MicroRNAs (miRNAs) are non-coding single-stranded small RNAs with 21~23 nucleotides, regulating the expression of target genes in a sequence-specific way (29). Recent studies have revealed that macrophage-derived exosomes carry miRNAs to mediate cell-to-cell communication, which contributes to the pathogenesis of many diseases (30). Feng Zhzh et al. found that miR-155-5p in TAM-derived exosomes heightens smooth muscle cell proliferation and migration by targeting Gremlin 1, thus promoting the activation and infiltration of intracranial aneurysm cells (31). MiR-5106 is highly expressed in M2-polarized macrophage-derived exosomes. It induces bone marrow mesenchymal stem cell osteogenic differentiation to accelerate fracture healing by targeting salt-inducible kinase 2 and 3. Meanwhile, local injection of miR-5106 agonist or M2D-Exos at the fracture site can accelerate healing *in vivo* (32). Deng FH et al. stated that miR-590-3p in M2-polarized macrophage-derived exosomes could transfer from macrophages to epithelial cells, promoting epithelial cell proliferation and wound healing. Mechanically, miR-590-3p targets large tumor suppressor kinase 1 (LATS1) and then activates the Yes-associated protein (YAP)/β-catenin pathway, thereby reducing inflammatory release, improving wound healing ability of epithelial cells, and alleviating ulcerative colitis (33). We first discovered that M2-polarized macrophages amplified CC cell proliferation, migration, invasion, and weakened apoptosis. Subsequently, we found that miR-183-5p was enriched in M2-TAM-Exo. Overexpressing miR-183-5p enhanced the malignant phenotype of CC cells, while inhibiting miR-183-5p exerted the opposite effect. The above results proved that M2-TAM-Exo aggravated CC through the interaction of miR-183-5p with CC cells.

Furthermore, by searching bioinformatics, we found that miR-183-5p accelerates CC by targeting THEM4 and activating AKT/NF-κB pathway. THEM4, also known as the C-terminal regulatory protein, plays an essential role in many tissues and cells. THEM4 can inhibit the phosphorylation of Akt and is an endogenous inhibitor of Akt (34, 35). In the past few years, emerging studies have been done on THEM4/AKT. Shin J-Y et al. found that in liver cancer mouse models, THEM4 hampers cell proliferation and angiogenesis by inhibiting the AKT phosphorylation, exhibiting anti-tumor effects (36). Multiple studies have confirmed the regulatory effect of Akt/NF-κB in CC. For example, the study of Adisan Ed et al. confirmed that diclofenac triggers the dephosphorylation of PTEN, PDK and AKT, thereby inactivating the PI3K/Akt pathway in HCT 116 CC cells and suppressing CC evolvement (37). C-X-C chemokine receptor type 7 contributes to CC cell growth and angiogenesis by inactivating the phosphorylation of Akt and ERK (38). Nevertheless, whether the THEM4/Akt axis plays a role in CC remains to be studied. Here, we found that THEM4 was targeted by miR-183-5p, and overexpressing THEM4 inhibited the malignant phenotype of CC. MiR-183-5p activated PI3K/AKT and NF-κB by targeting THEM4, thereby mediating CC progression.

Overall, our study manifested that exosomal miR-183-5p shuttled by M2-TAM plays a key role in CC. MiR-183-5p facilitates AKT/NF-κB pathway by targeting THEM4, thus promoting the proliferation, invasion and metastasis of CC cells. This article provides new therapeutic approaches for CC treatment.

## Data Availability Statement

The datasets presented in this study can be found in online repositories. The names of the repository/repositories and accession number(s) can be found in the article/supplementary material.

## Ethics Statement

The animal study was reviewed and approved by the Ethics Review Board of Beijing Chaoyang Hospital, Capital Medical University.

## Author Contributions

Conceived and designed the experiments: SZ, DL, ZW. Performed the experiments: SZ, MZ, FY, CS. Statistical analysis: CY, ZW. Wrote the paper: MZ, ZW, YL. All authors contributed to the article and approved the submitted version.

## Funding

National Natural Science Foundation of China (NSFC) (81672389, 81874063).

## Conflict of Interest

The authors declare that the research was conducted in the absence of any commercial or financial relationships that could be construed as a potential conflict of interest.
